# Supplementary material for: MetaQTL: a package of new computational methods for the meta-analysis of QTL mapping experiments
Source: BMC Bioinformatics. 2007 Feb 8;8:49. doi: 10.1186/1471-2105-8-49 (PMC1808479; doi:10.1186/1471-2105-8-49)
Supplement: Additional File 7 — MetaQTL Package : jar file and tutorial. This Zip archive contains both the MetaQTL JAR file and the files of the tutorial. [file 1471-2105-8-49-S7.zip › org.inra.metaqtl/doc/index-files/index-2.html]

B-Index


|  |  |  |
| --- | --- | --- |
| **Overview** \| Package \| Class \| Use \| **Tree** \| **Deprecated** \| **Index** \| **Help** | |  |
| **PREV LETTER**   **NEXT LETTER** | **FRAMES**    **NO FRAMES**     **All Classes** |  |


A B C D E F G H I K L M N O P Q R S T U V W X Y Z 

---


## **B**

**BACKGROUND\_COLOR** - Static variable in class org.metaqtl.graph.MetaGraphPar: The color of the backgroud. **base** - Static variable in class org.metaqtl.graph.PaletteColor: **BC** - Static variable in interface org.thalia.bio.IBioCrossTypes: **beta(double, double)** - Static method in class org.metaqtl.numrec.NumericalUtilities: Returns the beta function I(a,b) **betacf(double, double, double)** - Static method in class org.metaqtl.numrec.NumericalUtilities: Used by betai : Evaluates continued fraction for incomplete beta function by modified Lentz's method. **betai(double, double, double)** - Static method in class org.metaqtl.numrec.NumericalUtilities: Returns the incomplete beta function Ix(a,b) **BIC** - Static variable in class org.metaqtl.EMCriteria: **bic** - Variable in class org.metaqtl.EMCriteria: **BIC\_NAME** - Static variable in class org.metaqtl.EMCriteria: **BioCrossTypes** - Class in org.thalia.bio.util: **BioCrossTypes()** - Constructor for class org.thalia.bio.util.BioCrossTypes: **BioEntity** - Class in org.thalia.bio.entity: Class Description Here **BioEntity()** - Constructor for class org.thalia.bio.entity.BioEntity: **BioEntity(String, IBioEntity)** - Constructor for class org.thalia.bio.entity.BioEntity: **BioEntityAdapter** - Class in org.thalia.bio.entity.adapter: Class Description Here **BioEntityAdapter()** - Constructor for class org.thalia.bio.entity.adapter.BioEntityAdapter: **BioEntityBean** - Class in org.thalia.bio.entity.bean: Class Description Here **BioEntityBean()** - Constructor for class org.thalia.bio.entity.bean.BioEntityBean: **BioEntityCollection** - Class in org.thalia.bio.entity: Class Description Here **BioEntityCollection()** - Constructor for class org.thalia.bio.entity.BioEntityCollection: **BioEntityCollection(int)** - Constructor for class org.thalia.bio.entity.BioEntityCollection: **BioEntityCollectionAdapter** - Class in org.thalia.bio.entity.adapter: Class Description Here **BioEntityCollectionAdapter()** - Constructor for class org.thalia.bio.entity.adapter.BioEntityCollectionAdapter: **BioEntityCollectionBean** - Class in org.thalia.bio.entity.bean: Class Description Here **BioEntityCollectionBean()** - Constructor for class org.thalia.bio.entity.bean.BioEntityCollectionBean: **BioEntityContainer** - Class in org.thalia.bio.entity: Class Description Here **BioEntityContainer()** - Constructor for class org.thalia.bio.entity.BioEntityContainer: **BioEntityContainer(String, IBioEntity)** - Constructor for class org.thalia.bio.entity.BioEntityContainer: **BioEntityFactory** - Class in org.thalia.bio.entity.factory: Class Description Here **BioEntityFactory()** - Constructor for class org.thalia.bio.entity.factory.BioEntityFactory: **BioEntityNameComparator** - Class in org.thalia.bio.util: Class Description Here **BioLocusTypes** - Class in org.thalia.bio.util: **BioLocusTypes()** - Constructor for class org.thalia.bio.util.BioLocusTypes: **BioUtilities** - Class in org.thalia.bio.util: General comments must be added here. **BioUtilities()** - Constructor for class org.thalia.bio.util.BioUtilities: **BOX\_HEIGHT** - Static variable in class org.metaqtl.graph.GraphFactory: **BOX\_WIDTH** - Static variable in class org.metaqtl.graph.GraphFactory: **build(Graphics2D)** - Method in class org.metaqtl.graph.ChromLayer: **build(Graphics2D)** - Method in class org.metaqtl.graph.Layer: Build the layer. **build(Graphics2D)** - Method in class org.metaqtl.graph.QtlLayer: **build(Graphics2D)** - Method in class org.metaqtl.graph.QtlPartitionLayer: **build(Graphics2D)** - Method in class org.metaqtl.graph.QtlTreeLayer: **buildQtlTree(MetaQtlData)** - Method in class org.metaqtl.algo.QtlTreeAlgorithm: For the given data points this method build the tree which represents the hierarchical clustering. **buildShape(Graphics2D, Object)** - Method in class org.metaqtl.graph.ChromShape: **buildShape(Graphics2D, Object)** - Method in class org.metaqtl.graph.GraphShape: **buildShape(Graphics2D, Object)** - Method in class org.metaqtl.graph.QtlShape: This methods create a new QtlShape from the given graphics context.

---


|  |  |  |
| --- | --- | --- |
| **Overview** \| Package \| Class \| Use \| **Tree** \| **Deprecated** \| **Index** \| **Help** | |  |
| **PREV LETTER**   **NEXT LETTER** | **FRAMES**    **NO FRAMES**     **All Classes** |  |


A B C D E F G H I K L M N O P Q R S T U V W X Y Z 

---
